# Design and Tuning of Nanofluids Applied to Chemical Enhanced Oil Recovery Based on the Surfactant–Nanoparticle–Brine Interaction: From Laboratory Experiments to Oil Field Application

**DOI:** 10.3390/nano10081579

**Published:** 2020-08-11

**Authors:** Carlos A. Franco, Lady J. Giraldo, Carlos H. Candela, Karla M. Bernal, Fabio Villamil, Daniel Montes, Sergio H. Lopera, Camilo A. Franco, Farid B. Cortés

**Affiliations:** 1 Gerencia de Desarrollo Sur, Ecopetrol S.A., Neiva, Huila 410010, Colombia; carlosal.franco@ecopetrol.com.co (C.A.F.); carlos.candela@ecopetrol.com.co (C.H.C.); karlama.bernal@ecopetrol.com.co (K.M.B.); fabio.villamil@ecopetrol.com.co (F.V.); 2 Grupo de Investigación en Fenómenos de Superficie—Michael Polanyi, Facultad de Minas, Universidad Nacional de Colombia Sede Medellín, Kr 80 No. 65-223, Medellín, Antioquia 050034, Colombia; ljgiraldop@unal.edu.co (L.J.G.); damontespi@unal.edu.co (D.M.); 3 Grupo de Investigación en Yacimientos de Hidrocarburos, Facultad de Minas, Universidad Nacional de Colombia Sede Medellín, Kr 80 No. 65-223, Medellín, Antioquia 050034, Colombia; shlopera@unal.edu.co

**Keywords:** capillary number, CEOR, field trial, interfacial tension, nanofluids, surfactant flooding

## Abstract

The primary objective of this study is to develop a novel experimental nanofluid based on surfactant–nanoparticle–brine tuning, subsequently evaluate its performance in the laboratory under reservoir conditions, then upscale the design for a field trial of the nanotechnology-enhanced surfactant injection process. Two different mixtures of commercial anionic surfactants (SA and SB) were characterized by their critical micelle concentration (CMC), density, and Fourier transform infrared (FTIR) spectra. Two types of commercial nanoparticles (CNA and CNB) were utilized, and they were characterized by S_BET_, FTIR spectra, hydrodynamic mean sizes (dp_50_), isoelectric points (pH_IEP_), and functional groups. The evaluation of both surfactant–nanoparticle systems demonstrated that the best performance was obtained with a total dissolved solid (TDS) of 0.75% with the SA surfactant and the CNA nanoparticles. A nanofluid formulation with 100 mg·L^−1^ of CNA provided suitable interfacial tension (IFT) values between 0.18 and 0.15 mN·m^−1^ for a surfactant dosage range of 750–1000 mg·L^−1^. Results obtained from adsorption tests indicated that the surfactant adsorption on the rock would be reduced by at least 40% under static and dynamic conditions due to nanoparticle addition. Moreover, during core flooding tests, it was observed that the recovery factor was increased by 22% for the nanofluid usage in contrast with a 17% increase with only the use of the surfactant. These results are related to the estimated capillary number of 3 × 10^−5^, 3 × 10^−4^, and 5 × 10^−4^ for the brine, the surfactant, and the nanofluid, respectively, as well as to the reduction in the surfactant adsorption on the rock which enhances the efficiency of the process. The field trial application was performed with the same nanofluid formulation in the two different injection patterns of a Colombian oil field and represented the first application worldwide of nanoparticles/nanofluids in enhanced oil recovery (EOR) processes. The cumulative incremental oil production was nearly 30,035 Bbls for both injection patterns by May 19, 2020. The decline rate was estimated through an exponential model to be −0.104 month^−1^ before the intervention, to −0.016 month^−1^ after the nanofluid injection. The pilot was designed based on a production increment of 3.5%, which was successfully surpassed with this field test with an increment of 27.3%. This application is the first, worldwide, to demonstrate surfactant flooding assisted by nanotechnology in a chemical enhanced oil recovery (CEOR) process in a low interfacial tension region.

## 1. Introduction

Implementation of nanotechnology for improving the performance of multiple operations within the oil and gas industry, including enhanced oil recovery (EOR) techniques, is currently a major research focus [1,2,3,4]. The nanosized materials provide superb characteristics that enable the increase in crude oil recovery with outstanding profit margins [5,6].

In chemical enhanced oil recovery (CEOR) nanoparticles could be utilized alongside different fluid formulations [7,8], as nanoparticulated systems are able to increase the sweep efficiency by improving the capillary and/or viscous forces under reservoir conditions and releasing the trapped crude oil by altering/reverting the porous media wettability [9,10,11], decreasing the interfacial tension (IFT) between the aqueous phase and the crude oil [12,13,14], and increasing the displacement fluid viscosity [15,16,17].

Surfactant flooding is one of the most used CEOR techniques [18,19] for mobilizing the residual oil in the formation. This technique works by the interaction of the tensoactive agents with the aqueous phase/crude oil interphase, reducing the IFT values, and facilitating trapped oil mobilization during displacement-like operations [20,21]. Depending on the IFT value between the oil and the aqueous phases upon surfactant inclusion, these can be commonly considered within a low (3 mN·m^−1^ to 10^−2^ mN·m^−1^) or ultra-low (10^−2^ mN·m^−1^ to 10^−6^ mN·m^−1^) range [22,23,24]. At lower IFT values, the displacement sweep efficiency is improved due to the capillary pressure reduction [25,26] and the formation of a third phase in the zone near the brine/oil interphase, which is composed of an Oil-in-Water (O/W) micro-emulsion (e.g., Winsor type III) [27]. As this third phase has a higher viscosity than the brine used as the displacement fluid, the mobility ratio is improved as well as the sweep efficiency [28,29]. Thus, in CEOR applications it is typically used fluids formulations that produce ultra-low IFT (ULIFT) values. However, the maintenance of these IFT values under reservoir conditions could be challenging as the process performance could be extensively reduced due to the surfactant loss produced by its adsorption on the porous media [30,31,32,33,34]. In addition, surfactant interaction with the aqueous phase/crude oil interphase could be hindered, which further reduces the technique’s cost-effectiveness [35,36]. Moreover, the implementation of surfactant flooding at the low IFT region could be enough to increase the crude oil recovery under certain reservoir conditions such as oil saturation, brine composition, and capillary forces, among others [37]. This increased recovery is mainly due to the low costs of the technique, which favors the application in field tests based on the surfactant loss due to the adsorption phenomena [32].

Several surfactant flooding enhancement approaches utilizing nanotechnology have been developed to overcome the conventional process limits and issues associated with adsorption activity and hence, extend the technique profit margin [38,39]. Among the most notable of these techniques are those used for ULIFT applications. Although there have been no reports of nanotechnology-assisted surfactant adsorption reduction under reservoir or field conditions, and without overcoming these challenges, the application of this technology would be more expensive than conventional surfactant flooding, a few studies have reported that a large amount of nanoparticles was needed to reach a slight change in IFT [40]. Thus, the surfactant application that functions in the low IFT (LIFT) region, which can be more economical than the ULIFT process is equally important.

Several authors have worked on the topic of LIFT with nanomaterials. Suleimanov et al. [41] formulated a nanofluid with non-ferrous metal oxide nanoparticles and an anionic surface-active agent and evaluated IFT changes upon nanomaterial inclusion in the dispersion through the pendant drop technique. They used surfactant dosages of 0.0078 and 0.05 wt% and determined that with just 0.001 wt% of nanoparticles, the IFT was reduced by 70–90%, down to values of ~1 mN·m^−1^, compared to the usage of the surfactant only. In addition, Zargartalebi et al. [42] evaluated partially hydrophobic and hydrophilic silica (SiO_2_) nanoparticles for enhanced surfactant flooding at a fixed concentration of 2000 mg·L^−1^. The authors obtained IFT values of 2 mN·m^−1^ and increased the original oil in place (OOIP) recovery by more than 5%. Subsequently, Zhao et al. [43] evaluated the synergy between silica nanoparticles and surfactants in EOR applications by spontaneous imbibition experiments using a concentration of 0.1 wt% for both components. The authors determined that recovery enhancement was related to the improvement of the capillary forces of the displacement fluid, such as the IFT, which they reduced from 21 mN·m^−1^ to 1.2 mN·m^−1^. Cortés et al. [44] synthesized nanocapsules for controlled delivery of encapsulated surfactants with two different types of these tensoactive agents, namely Span 20 and Petro 50. The authors concluded that the surfactant adsorption on the rock was negligible, while the IFT was reduced up to 0.15 mN·m^−1^ with just 10 mg·L^−1^ of the material. Betancur et al. [45] evaluated the interactions between silica gel nanoparticles and a cationic surfactant for the reduction in the IFT without considering the brine composition effect. The tests were conducted at nanoparticles and surfactant dosages of 10–1000 and 0–8000 mg·L^−1^, respectively. The results showed an IFT reduction of up to 3 mN·m^−1^, which is in the low IFT region. Finally, Rezaei et al. [46] evaluated silica nanoparticles in a CEOR process with an amphoteric surfactant, namely cocamidopropyl betaine (CAPB). The IFT was reduced to 1 mN·m^−1^ at a nanoparticle dosage of 1000 mg·L^−1^, and the OOIP recovery was improved up to 12.2%.

Although there have been multiple studies on surfactant flooding enhancement with nanomaterials in both ULIFT and LIFT applications, there have been few insights on appropriate upscaling from the laboratory to a relevant field environment. Betancur et al. [47] studied the effect of nanofluids on surfactant–nanoparticle–brine interactions with the goal of developing an experimental methodology for a ULIFT application. The authors evaluated three different preparation method sequences: (I) salts–nanoparticles–surfactants, (II) salts–surfactants–nanoparticles, and (III) surfactant–nanoparticles–salts. The authors concluded that the dissolved ions play an important role in the surfactant–surfactant interaction and subsequent micelle formation, which enhances the tensoactive agent interaction with the brine/oil interphase. In this sense, the method sequence II provided the greater nanofluid performance, as surfactant addition followed by nanomaterials did not restrict the ion availability, and the surfactant was thereby able to form micelles and freely interact with the nanoparticle surfaces and the aqueous phase/crude oil interphase. No similar studies have been reported investigating a LIFT application [48].

Considering the advances in nanotechnology usage for the enhancement of the surfactant flooding operations based on the laboratory tests at steady-state and reservoir conditions, additional evaluations are needed to advance a LIFT-nanoparticles-assisted application at a field trial. In this regard, the additional experimentation must consider both the surfactant–nanoparticle–brine interactions as well as the fluid–rock interactions and the changes in the capillary forces. This last qualification would provide additional insights into appropriate surfactant–nanoparticle–brine tuning for a LIFT application. It would also help to reduce surfactant loss due to its adsorption on the rock and therefore increase the cost-effectiveness of the process. Thus, the main objective of this study is to develop an experimental design utilizing surfactant–nanoparticle–brine tuning under reservoir conditions based on static and dynamic tests. This design allows for scale-up to a field trial application of an enhanced surfactant injection process through nanotechnology and enables optimal operation cost-effectiveness.

This study describes the experimentation conducted to enable upscaling and field testing of the intervention in a Colombian field, which produces a near-intermediate heavy crude oil (19°API). The manuscript is divided into three parts: (i) static tests based on fluid-fluid and fluid-rock interactions to characterize the surfactants (CMC, FTIR) and nanoparticles (S_BET_, dp50, FTIR, pH_IEP_) and their mix by IFT, adsorption experiments, contact angle, and imbibition tests; (ii) core flooding tests under reservoir conditions, and (iii) the first known field trial for nanoparticle/nanofluid injection in EOR processes. The nanofluid flooding was performed in two different injection patterns (patterns A and B), which were characterized in terms of the injector wells connectivity with their surrounding production wells. The surfactant flooding process was intended for a low IFT application; thereby, the experimentation was focused on this particular purpose. This novel approach will open a broader landscape on the benefits of nanotechnology in EOR applications and provide a powerful tool for the massification of these emerging technologies.

## 2. Materials and Methods

### 2.1. Materials

For the laboratory experimentation, including the surfactant–nanoparticle–brine tuning, two different mixtures of commercial anionic surfactants (SA and SB) with HLB (hydrophilic–lipophilic balance) values of 11 units, and nanoparticles (CNA and CNB) were provided by Petroraza S.A.S (Medellín, Colombia). Synthetic brine was formulated to resemble the composition of the on-site injection brine. It included the salts NaCl, CaCl_2_.2H_2_O, MgCl_2_.6H_2_O, BaCl_2_.2H_2_O, and KCl (Sigma Aldrich, St. Louis, MO, USA); their respective concentrations (g·L^−1^) are reported in Table 1.

Furthermore, a Colombian heavy crude oil with 19° API sampled from the field of study was utilized in the tests. The SARA composition of the oil showed that the saturates, aromatics, resins, and asphaltenes composition was 29.4%, 35.6%, 26.8%, and 8.2%, respectively. API gravity measurement was conducted following the ASTM D-1250 standard, while SARA analysis followed the IP 469 standard using a TLC-FID/FPD Iatroscan MK6 (Iatron Labs Inc, Tokyo, Japan).

### 2.2. Methods

Selection and tuning of the surfactant–nanoparticle couple in brine to determine optimal dosage were carried out by basic characterization of each component and performance testing in fluid–fluid and fluid–rock interactions, including IFT, wettability, and adsorption tests. Then, the oil recovery was tested by coreflooding on a laboratory-scale under reservoir conditions. Finally, the upscaling to a trial in a Colombian field was developed using an injection system in two flow patterns of injector-producing wells. Field trial results were evaluated in terms of incremental production. For tuning the surfactant–nanoparticle–brine, first, an initial optimal dosage of surfactants SA and SB in the synthetic brine formulation was selected, then the total dissolved solid (TDS) was adjusted to ensure the best behavior of the chemical surfactant. Finally, to the optimal concentrations of surfactant and TDS, nanoparticles were added in different dosages, selecting and readjusting each concentration according to the dispersion behavior to assure the best surfactant–nanoparticle performance.

#### 2.2.1. Surfactant and Nanoparticle Characterization

The surfactants and nanoparticles were characterized separately by Fourier transform infrared spectroscopy to determine their functional groups [49] (IRAffinity-1 FTIR spectrometer, Shimadzu, Japan). The surfactant properties, such as critical micelle concentration (CMC) and density, were also measured. The CMC was evaluated by varying the surfactant concentration in the brine and using the conductivity and surface tension of the dispersions as response variables with the slope change indicating the CMC value [50,51,52,53]. Conductivity was measured using a pH Orion Star™ A211 (Thermo Scientific™, Waltham, MA, USA), while surface tension tests were conducted using a K9-MK1 (KRÜSS GmbH, Germany) tensiometer with the Du Nouy ring method [54].

The nanoparticles were characterized by their size (dp50), their hydrodynamic diameter determined in an aqueous phase using a NanoPlus-3 (Micromeritics, GA, USA) through the dynamic light scattering (DLS) technique, and their surface area (S_BET_) through the Brauneur–Emmett–Teller method [55] by N_2_ physisorption at −196 °C using an Aurosorb-1 Quantacrome (USA). Their isoelectric point of net charge zero (pH_IEP_) was measured using the NanoPlus-3.

#### 2.2.2. Surfactant–Nanoparticle–Brine Tuning

**Figure 1:**
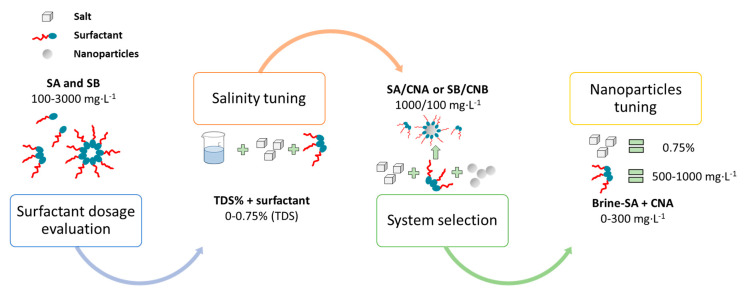
Surfactant–nanoparticle–brine tuning scheme.

Interfacial tension (IFT) measurements were conducted for tuning of the surfactant–nanoparticle–brine to guarantee improved performance in the EOR process. First, each surfactant sample was evaluated individually in the formulation of synthetic brine at concentrations between 100 and 3000 mg·L^−1^ of SA and SB, with the IFT as the response variable for a preliminary selection of the best surfactant in their initial optimal dosage. As the salinity or total dissolved solids (TDS) is one of the variables with a major effect on tuning, TDS for the brine preparation was conducted on each surfactant at a fixed concentration selected previously in the first stage. The TDS content was further varied (0.00%, 0.22%, 0.37%, 0.52%, and 0.75%) using the brine formulation (Table 1) as a stock solution. The TDS with the lowest IFT value was chosen for further experimentation. Subsequently, the surfactants were tested with a fixed concentration (100 mg·L^−1^) of nanoparticles to serve as an initial approach of studying nanoparticle inclusion in the modification of the interfacial balance with the surfactant. In the tuning of the surfactant–nanoparticle–brine mixture, it is also important to consider the possible readjustment of the surfactant concentration as a major benefit. The effect of surfactant dosage was evaluated (500, 750, and 1000 mg·L^−1^) as well as the nanoparticle concentration (0, 50, 100, and 300 mg·L^−1^). Figure 1 presents a summary of the tuning experimentation.

The surfactant dosage was maintained above its respective CMC value throughout the experimentation to ensure interfacial dynamic equilibrium with the lower values of IFT and assuming its possible loss due to adsorption phenomena on the rock surface, which would be necessary for further evaluations. The aqueous dispersions were prepared following the order of brine–surfactant–nanoparticle as reported in previous studies [47], to avoid interactions of the dissolved ions with the nanoparticles rather than with the surfactants, which may hinder micelle formation and the IFT reduction due to the active interaction of these with the brine/oil interphase [56,57].

The IFT tests were developed using a spinning drop M6500 (Grace Instrument, United States) tensiometer. A crude oil droplet was added to the respective aqueous dispersions, and the value was determined when the system reached the equilibrium per the Vonnegut equation [58,59]:

(1) $$\gamma =1.44e-7.\Delta \rho .D^{3}.\theta^{2}$$

in which Δρ (g·mL^−1^) is the density difference of the fluids, θ (rpm) is the angular velocity, D (mm) is the diameter of the oil phase droplet, and γ (mN·m^−1^) is the IFT between the aqueous dispersion and the crude oil. All the measurements were performed at reservoir temperature (50 °C) in triplicate presented with standard error bars.

#### 2.2.3. Batch Adsorption Tests

Adsorption isotherms were constructed to determine the surfactant loss to adsorption on the rock in the absence of the nanoparticulated systems varying the surfactant concentration from 500 to 1000 mg·L^−1^. Ottawa sand grains were used as the adsorbent. The adsorption isotherms were constructed at reservoir temperature following the same mixture pattern of the IFT experiments in which the surfactant was added to the previously prepared brine, and then the adsorbents were included. The dispersions were stirred for 24 h at 200 rpm. The adsorptive capacity of the adsorbents was assessed as described in previous studies [45,47], based on a thermogravimetric method. The sole adsorbents were subjected to heat from 30 to 800 °C with a temperature increase of 5 °C·min^−1^ under a dried air atmosphere using a TGA analyzer (Q50, TA Instruments Inc., New Castle, DE, USA) to obtain the mass decomposition target. The process was repeated for the materials with the adsorbed surfactant, and from the registered mass loss, the target was subtracted. The adsorption of the surfactant over the surface of the nanoparticle was evaluated following the same procedure for adsorbent dosages from 50 to 300 mg·L^−1^, which resembles the conditions of the IFT measurements. In all cases, the experiments were conducted in triplicate to ensure reproducibility. Moreover, the amount of surfactant adsorbed over the nanoparticle’s surface was estimated as follows:

(2) $$N_{ads}=\frac{\left(C_{i}-C_{E}\right)}{M}$$

where $C_{i}$ (mg·L^−1^) and $C_{E}$ (mg·L^−1^) are, respectively, the initial concentration of the surfactant in solution and the equilibrium concentration of surfactant; M(g·L^−1^) is the mass ratio of the nanoparticles and solution volume. Alongside this, the solid–liquid equilibrium (SLE) model was used to describe the adsorption isotherms of the surfactant onto the nanoparticles and the rock. It consists of a model developed by Montoya et al. [60] related to the adsorption of self-assembly molecules such as surfactants on the nanoparticle’s surface [8,45]. The model is described as follows:

(3) $$C_{E}=\frac{\psi H}{1+K\psi }exp\left(\frac{\psi }{N_{m}\cdot A}\right)$$

(4) $$\psi =\left(-1+\sqrt{1+4K\cdot \xi }\right)$$

(5) $$\xi =\left[N_{m}\cdot Nads/\left(N_{m}-Nads\right)\right]A$$

where $N_{m}$ (mg·m^−2^) is the maximum adsorption capacity, A (m^2^·mg^−1^) is the surface area of the nanoparticles measured through the BET method, K (g·g^−1^) is a constant related to the adsorbate–adsorbate interaction after the formation of a surfactant monolayer on the nanoparticle surface, and H(mg·g^−1^) is the Henry’s constant related to the adsorbent–adsorbate affinity. The accuracy of the model was determined by root square mean error (RSME%).

#### 2.2.4. Rheological Measurements

The rheological behavior in terms of the viscosity vs. sear rate was determined for the brine and the dispersions in the absence and presence of the respective tuned dosages of nanoparticles. The flow curves were obtained using a Kinexus Pro (Malvern Instruments, Worcestershire, UK) rheometer equipped with a solvent trap and a Peltier cell for controlling the temperature with a precision of 1 × 10^−2^ °C. A concentric cylinders geometry (double GAP) was employed for carrying out the tests, ideal for these types of materials due to their low viscosity. An increasing shear rate ramp between 3 and 100 s^−1^ was used to perform the analysis at a temperature of 50 °C, as described in previous studies [12,61,62].

#### 2.2.5. Contact Angle Measurements

The contact angle was determined to ascertain the affinity of a surface to be wetted by a specific fluid. In this case, a rock surface affinity towards the water was determined. An oil wettable condition was created by immersing outcrops in an oily phase and encouraging asphaltene precipitation and deposition on its surface by the addition of *n*-heptane in a volume ratio of 30:70 to crude oil [9]. The outcrops were then immersed for 48 h in the brine and the dispersion systems in the absence and presence of nanoparticles. The cores were dried at 100 °C for 24 h. Finally, the contact angle for the water/air/rock system was estimated at 50 °C using a Theta optical tensiometer (Biolin Scientific, Sweden) equipped with a high definition camera. The outcrop was placed in the equipment platform, and a single droplet was released onto the sample’s surface. The camera recorded the deformation of the droplet, and the software calculated its contact angle. More information about this methodology can be found in our previous studies [9,12,63,64].

#### 2.2.6. Capillary Number Estimation

The capillary number (Nc) is a dimensionless number that is narrowly related to the efficiency sweep and further heavy oil recovery during displacement-like EOR methods [65,66,67]. This number relates to different properties associated with the oil displacement efficiency and recovery, such as the flow velocity, contact angle, displacement fluid viscosity, and IFT. Calculating this factor provides insights into the mechanisms driving the EOR process for specific fluids and reservoir properties, such as its mineralogy. Hence, to increase the performance of the displacement method, the Nc should be maximized [68]. The Nc is estimated using the following equation [69]:

(6) $$Nc=\frac{v.\mu }{\sigma .\cos\theta }$$

where μ (Pa∙s) is the viscosity of the displacement fluid, v (m∙s^−1^) is the average fluid velocity, σ (N∙m^−1^) is the IFT, and θ is the contact angle related to the wettability of the porous media [70].

#### 2.2.7. Coreflooding Tests

Although the materials are of nanometric size, and no blockage would be expected after their implementation under dynamic conditions [71], the surfactant adsorption on nanoparticle surfaces increases the entire assembly size, which could slightly affect the experiments. As preparation for crude oil recovery, injectivity tests were developed under the specific reservoir conditions (50 °C, and 9.31 MPa, and 3.45 MPa of overburden and back pressure, respectively) to ensure no porous blockage was generated by nanofluid injection. The procedure consisted of injecting 50 porous volumes (PVs) of crude oil followed by 100 PVs of surfactant and nanofluid separately to estimate the pressure drop (ΔP). The procedure is based on one described in our previous studies [12,71].

**Figure 2:**
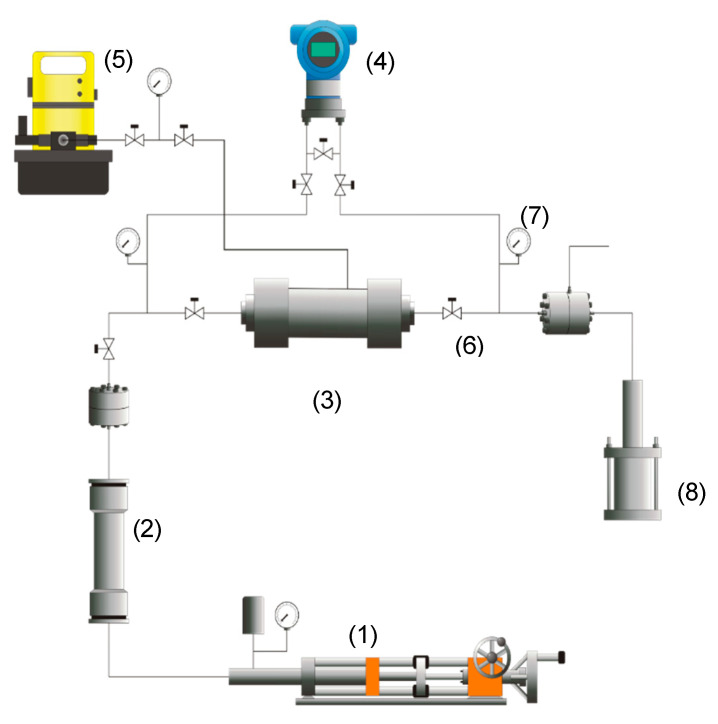
Schematic representation of the experimental setup for the displacement tests: (**1**) displacement pump, (**2**) cylinder, (**3**) sample core holder, (**4**) pressure transducer, (**5**) hydraulic pump, (**6**) valves, (**7**) manometers, and (**8**) a pressure multiplier.

In addition, crude oil recovery tests were conducted under reservoir conditions and using synthetic cores with the reservoir mineralogy. Two cores were used, one for the surfactant only and another for the nanofluid injection. The core properties are reported in Table 2. The porous media were saturated with 10 PVs of the brine until the same pressure drop was achieved, and 10 PVs of crude oil were injected to obtain a residual water condition (Swr), then 10 PVs of brine were injected again to determine the recovery factor baseline. It was ensured that the additional brine injection would not increase the recovery factor, and the pressure drop remained constant at the end of this stage. The porous media were prepared for the injection of each treatment with 5 PVs of crude oil, and 0.3 PVs of the surfactant or the nanofluid were injected. Finally, 10 PVs of brine were injected to determine the final recovery factor. Figure 2 shows the schematic representation of the setup used for the displacement test described above.

#### 2.2.8. Field Test

**Figure 3:**
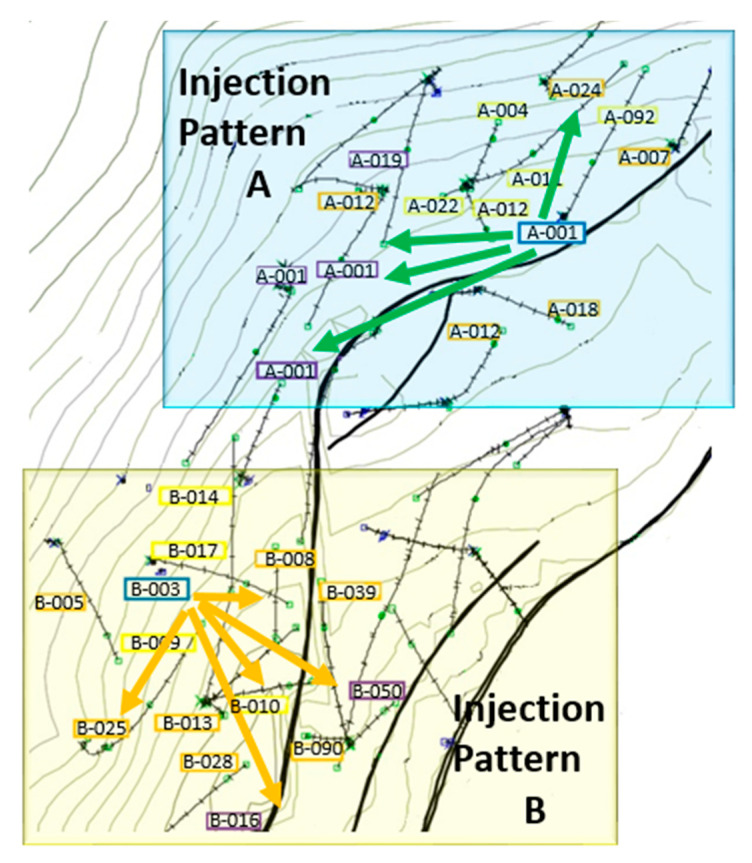
Representation of the flow injection patterns A and B in the Colombian oil field of interest.

An intervention with tracers was conducted in the oil field of interest to identify the connectivity and injection patterns between two injection wells and their surrounding production wells (Patterns A and B). Pattern A was composed of four production wells surrounding the injector and an additional 10 wells located at an outer periphery, which were part of the main pattern. Meanwhile, for Pattern B, there were five adjacent wells connected to the injector and an additional 10 wells in an outer periphery which related to the main pattern. A representation of the spatial and geographic distribution of Pattern A and Pattern B is shown in Figure 3.

After primary identification of the injection pattern connectivity of A and B, the nanofluid injection started on December 6, 2019, through both injection wells. The treatment was initially composed of 1000 and 100 mg∙L^−1^ of the SA surfactant and CNA nanoparticles, respectively, as described in our laboratory experiments. The surfactant dosage was then gradually decreased to maintain incremental production with a higher cost-effective margin. The surfactant concentration reductions are described in Table 3 for both patterns. Towards the final stage of the field test, the surfactant concentration was reduced to 400 and 350 mg∙L^−1^ for patterns A and B, respectively (Table 3). The SA concentration was maintained above its CMC value to guarantee its interaction with the aqueous phase/crude oil interphase, as a mass loss of the tensoactive agent due to its adsorption on the rock surface was still expected. The nanofluid injection was suspended on 14 April, 2020, to evaluate the pilot results, yet its effect was still observed on 19 May, 2020.

## 3. Results

The results are reported in six main sections: (I) materials characterization, (II) surfactant–nanoparticle–brine tuning, (III) nanoparticle effect on surfactant adsorption, (IV) capillary number estimation, (V) core flooding tests, and (VI) the field test.

### 3.1. Materials Characterization

#### 3.1.1. Surfactants

Density, CMC, and hydrophilic–lipophilic balance (HLB) (Table 4) measurements of SA and SB demonstrated that they have similar properties and are slightly different in CMC values. In this regard, further experimentation will increase surfactant concentrations above the CMC values, which is especially important for the contact angle and core displacement test in which there could be mass loss of the surfactant due to its adsorption on the rock surface [31,33,35]; thus, a surfactant concentration above the CMC is needed to guarantee the complete saturation of the brine/water interphase and obtain good performance during the IFT reduction. Furthermore, the HLB value of ~11 indicates that both surfactant mixtures are predominantly hydrophilic.

**Figure 4:**
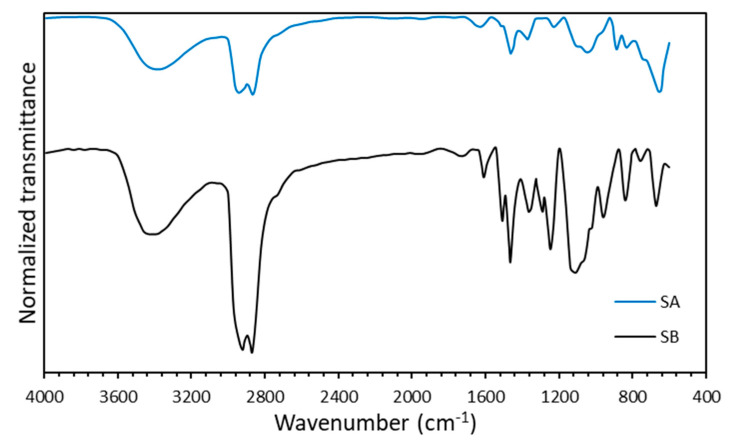
FTIR spectra obtained for the SA and SB surfactants.

The FTIR spectra (Figure 4) exhibiting the surfactant functional groups showed a similar composition with common bands for both surfactants but with variable intensity (transmittance). The wide band between 3650 and 3100 cm^−1^ was associated with O–H bonding [72,73]. The near band between 3000 and 2720 cm^−1^ corresponded to C–H bond vibrations, while the peaks between 1500–1190 cm^−1^ included C=C, C≡H, and C≡N related compounds. The peak centered in the region of 1190 and 860 cm^−1^ was usually attributed to polar species such as PO(OR)_3_ and -SO_2_-OH. The bands at 1245–1180 cm^−1^ corresponded to sulfonate stretching, confirmed by the peaks at 1050–1100 cm^−1^. Finally, the region between 800 and 600 cm^−1^ could be associated with P=C, -PO(OR)_2_, S=O, and aromatic ring vibrations [74]. Comparatively, SA and SB had slight differences in their FTIR spectra with an increase in intensity (decrease in transmittance) in the regions related to aromatic, sulfonate, and polar species between 600–1190 cm^−1^, and the aliphatic compounds in the bands of 2700–3000 cm^−1^, with an increase in intensity in both regions for surfactant SB.

#### 3.1.2. Nanoparticles

The CNA and CNB nanoparticle characterization in terms of their hydrodynamic size (dp_50_), surface area (S_BET_), and isoelectric point (pH_IEP_) are reported in Table 5. The evaluated nanomaterials had similar hydrodynamic sizes with different surface area values. Moreover, the isoelectric point of both indicated that these would maintain good stability at neutral or higher pH values. In this regard, as the brine pH was 7, a stable nanoparticle suspension would likely be formed, preventing their agglomeration and facilitating their interaction with the dispersed surfactant [75].

**Figure 5:**
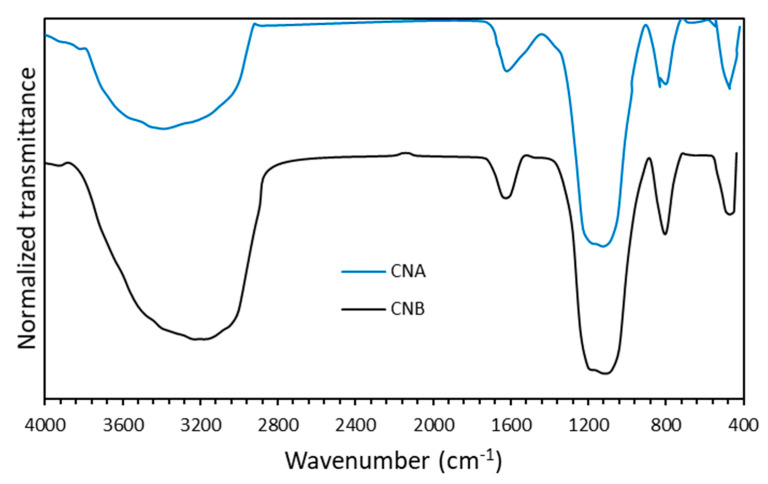
FTIR spectra obtained for the two types of commercial nanoparticles (CNA and CNB).

The FTIR spectra of the nanoparticles are presented in Figure 5. As mentioned above, the wide band between 3650 and 3100 cm^−1^ correlated with O–H bonding, which in this case represented the Silanol Si–OH groups [61,76]. Moreover, the peak centered at 1600 cm^−1^ corresponded to the O–H scissoring [77], while the pronounced peak between 1000 and 1300 cm^−1^ denoted the asymmetric stretching of the O–Si–O bonds [49,78]. The adjacent band, located between 720 and 880 cm^−1^, corresponded to the O–Si–O stretching vibration, and the bands at approximately 400 and 600 cm^−1^ represented the Si–O bond flection. These results indicated that the evaluated commercial nanoparticles were composed predominantly of SiO_2_.

### 3.2. Surfactant–Nanoparticle–Brine Tuning

As the experimentation carried out in this work is to ensure ideal upscaling for the implementation of the developed technology in a field test, the surfactant–nanoparticle–brine tuning is of particular interest to provide the best possible performance with representative fluids of the evaluated oil field. To this end, the salinity (TDS), surfactant, and nanoparticle types and their respective concentrations were evaluated, with the IFT as the response variable.

#### 3.2.1. Salinity Effect on IFT

Salinity, represented by TDS, is an important parameter to control during surfactant flooding processes. In every brine/oil system, in the presence of a particular tensoactive agent, there is an optimum salinity content ensuring micelle formation and dynamic adsorption on the interphase that enables a decrease in the IFT and the further formation of the microemulsion phase responsible for the displacement enhancement [79,80].

**Figure 6:**
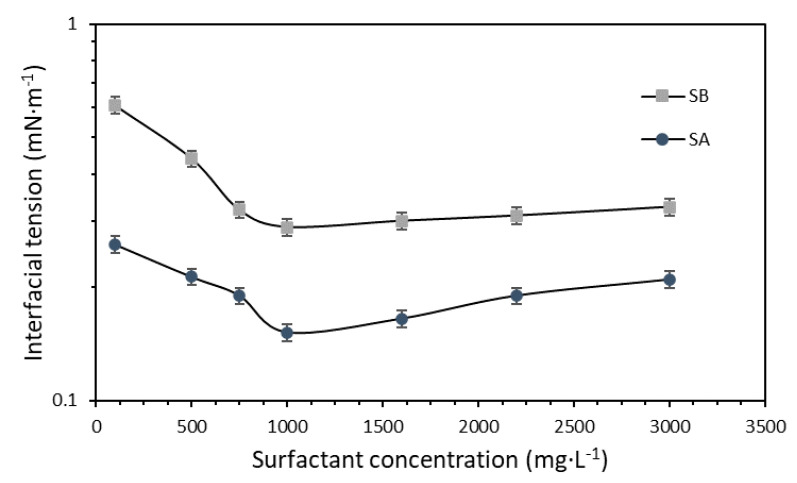
Interfacial tension measurements for surfactants SA and SB in synthetic brine. The tests were conducted at 50 °C, varying the surfactant dosage between 100 and 3000 mg·L^−1^. The symbols represent the experimental data, and the error bars correspond to their standard deviation.

The initial assessment of the IFT for surfactants SA and SB in brine formulation (Table 1) at dosages between 100 and 3000 mg·L^−1^ (Figure 6) demonstrated a clear trend of the individual performance of each surfactant. Surfactant SA achieved a greater decrease in IFT than SB in the initial approximation for the selection tuning of the dispersions. However, the lowest IFT values were achieved at 1000 mg·L^−1^ for both surfactants.

The dissolved ions in the aqueous phase interact with the polar heads of the surfactant molecules [81]. This interaction changes the charges of the molecules and reduces the repulsive forces between them. Thus, the surfactant–surfactant interactions are improved, and the formation of micelles is facilitated [82]. This phenomenon also enables easier saturation of the aqueous phase/crude oil interphase with the surfactant molecules, which results in an IFT decrease at lower surfactant dosages [37,83]. We studied the effect of TDS on the brine/oil IFT by varying its content from 0% to 0.75% using the brine formulation (Section 2.1) stock solution and with a fixed (separate) SA and SB concentration of 1000 mg·L^−1^ based on the IFT assessment (Figure 6).

**Figure 7:**
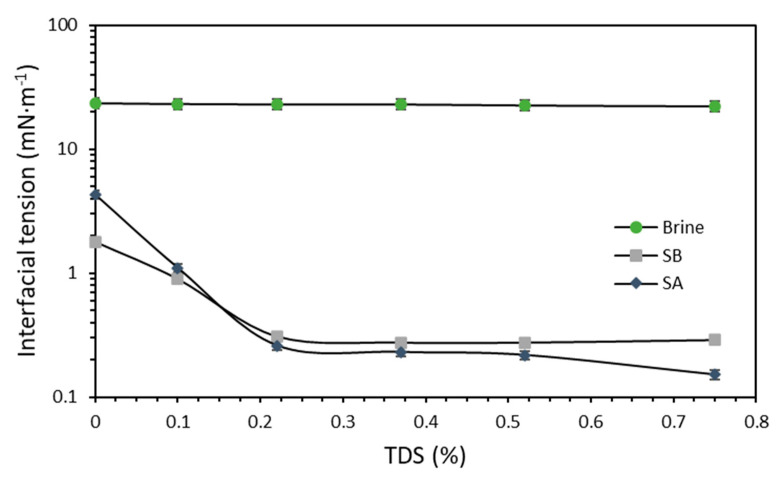
Interfacial tension measurements in brine total dissolved solid (TDS). The tests were conducted at 50 °C at a fixed concentration of 1000 mg·L^−1^ for each surfactant (SA and SB). The symbols represent the experimental data, and the error bars correspond to their standard deviation.

For almost all the evaluated TDS except 0%, the surfactant SA performed better than SB (Figure 7). This result would suggest that SA has a better affinity for the dissolved ions in the medium than SB, which would enhance its performance in the presence of the salts used for the brine formulation [45,47,84]. These results are consistent with the IFT assessment trend (Figure 6). Moreover, at least for the SA surfactant, the increase in the TDS accompanied a decrease in the measured IFT, with a value of 0.15 mN·m^−1^ at 0.75% TDS. For SB, a similar trend was observed with a slight increase in IFT at the same TDS with an IFT value of 0.28 mN·m^−1^. Both surfactants can reduce the IFT to values lower than 1 due to their hydrophilic–lipophilic balance (HLB) value, which is near 11 [85]. As a result, the TDS of 0.75% will be used in further experiments.

Another evaluation was conducted using the fixed amount of 0.75% TDS in the brine and with a constant concentration of 1000 and 100 mg·L^−1^ for the surfactants and the nanoparticles, respectively. The tests were performed to determine the best surfactant/nanoparticle combination for developing further tuning experiments. Notably, the dispersion preparations followed the order of brine–surfactant–nanoparticles to avoid undesired nanoparticle–ion interactions, which reduced the micelle formation of the surfactant and its interaction with the brine/oil interphase [56,57], as reported in other studies [47].

**Figure 8:**
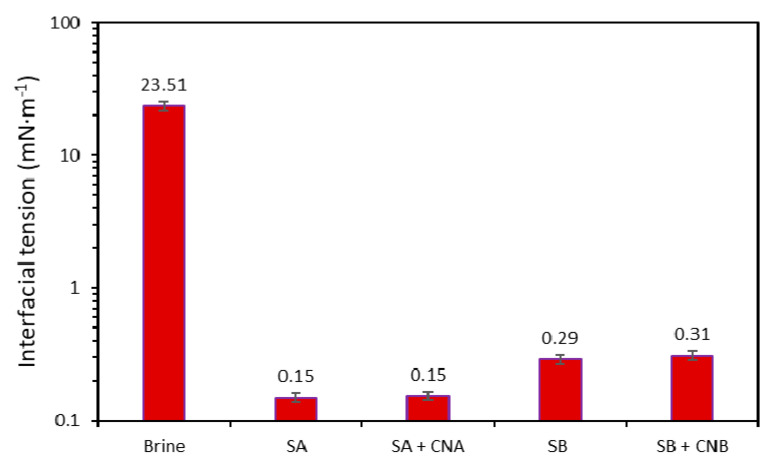
Interfacial tension measurements at a constant brine TDS of 0.75%. The tests were conducted at 50 °C at a fixed concentration of 1000 and 100 mg·L^−1^ for each surfactant (SA and SB) and nanoparticle (CNA and CNB), respectively. The standard deviation is shown as error bars.

From the graph (Figure 8), it was observed that the IFT for the brine/oil system was substantially reduced with the use of the dispersions. Although, with nanoparticle inclusion, there was a slight increase in the IFT values. This slight IFT increase is likely related to surfactant adsorption on the surface of the nanoparticles [45,84], which can confer other advantages such as wettability or viscosity to favor an increase in oil recovery. The effect was less visible for the SA/CNA combination, which exhibited a synergistic behavior, as it had almost the same value as the respective dispersion in the absence of nanoparticles. In contrast, the IFT value of the SB/CNB system increased from 0.28 to 0.31 mN·m^−1^.

The best performance for the mix of SA and CNA components could be attributed to the compositional differences mainly for the surfactant mixtures, which were represented by the intensity changes in the FTIR spectra (Figure 4). These intensity changes were related to the content of the hydrophilic or lipophilic groups, which favor the synergy between nanoparticles and surfactants. For SA, the functional groups C–H located from 2800 to 3000 cm^−1^ were of a lower intensity than for SB, suggesting short chains of aliphatic groups which may increase the dipole moment of surfactant molecules and decrease the steric effects that restrict nanoparticle interactions with the sulfonated groups of the surfactants (1180–1245 cm^−1^ bands). As previously reported [86,87], an increase in hydrocarbons chain is directly related to hydrophobic character and further solubility in the oil phase disfavoring the balance of micelle formation in the interphase. In energetic terms, the increase in hydrophobicity reduces the enthalpy and increases the entropy of the process affecting the interfacial adsorption [88]. Thus, SA has a better ability to saturate the oil–water interphase than SB.

Functional group analysis is an initial approximation of the interaction between the surfactant mixtures and nanoparticles, which will be further discussed in the following sections describing the adsorption phenomena.

Due to this synergistic process and its superior performance for reducing and/or maintaining the IFT, the SA/CNA combination was chosen to continue with the tuning process.

#### 3.2.2. Surfactant and Nanoparticles Dosage Evaluation

**Figure 9:**
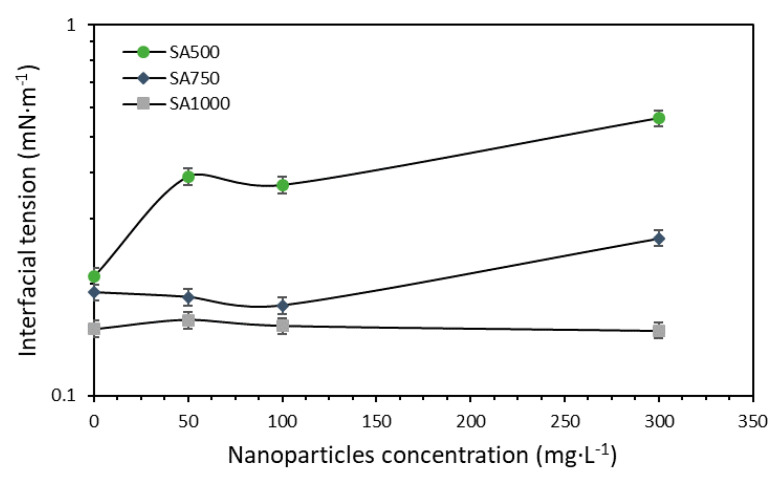
Interfacial tension measurements in constant brine TDS of 0.75%. The tests were conducted at 50 °C with SA surfactant and CNA nanoparticle concentrations from 500 to 1000 mg·L^−1^ and from 0 to 300 mg·L^−1^, respectively. The symbols represent the experimental data, and error bars represent standard deviation.

The nanofluid tuning process was continued with a constant brine TDS of 0.75% and varying the SA surfactant and CNA nanoparticle concentrations from 500 to 1000 mg·L^−1^ and from 0 to 300 mg·L^−1^, respectively. The results are reported in Figure 9, and the SA500, SA750, and SA1000 captions correspond to the surfactant dosages of 500, 750, and 1000 mg·L^−1^. For surfactant concentrations near their CMC, the addition of nanoparticles tended to increase the IFT rather than reduce or maintain it. This result is explained by the surfactant adsorption onto nanoparticle surfaces, which reduces the availability of the tensoactive agent and hence its interaction with the aqueous phase/crude oil interphase [13,84]. These results are consistent with [45,84], in which no reduction in IFT was observed in the presence of nanoparticles under specific concentrations. Thus, this effect should be reduced by increasing the surfactant concentration in the brine, which is reflected in the SA750 and SA1000 experimental data. For a SA surfactant concentration of 750 mg·L^−1^, the IFT was reduced with nanoparticles present until a CNA dosage of 300 mg·L^−1^. On the other hand, at a concentration of 1000 mg·L^−1^ of the tensoactive agent, the IFT was slightly reduced or maintained for all the evaluated nanoparticle concentrations. This result indicates that there is a synergistic process for the SA/CNA system for this last concentration, in which the free surfactant is able to form micelles, enhancing its interaction with the aqueous phase/crude oil interphase and supported by the surfactant adsorbed onto nanoparticle surfaces [47].

Considering these results and for practicality during the displacement tests and the field trial, a concentration of 100 mg·L^−1^ of the CNA nanoparticles was chosen to conduct additional tests, as it is suitable for an IFT reduction/maintenance for various surfactants concentrations rather than just for the single 1000 mg·L^−1^ of SA. The last result would be of interest while conducting the field test, as the surfactant concentration for the EOR process would be gradually reduced to reach a suitable cost-effective margin. It is worth noting that the main advantages of the nanoparticles during a surfactant flooding process lie in the inhibition of the surfactant adsorption over the rock surface [40,41,47].

### 3.3. The Effect of Nanoparticles on Surfactant Adsorption

**Figure 10:**
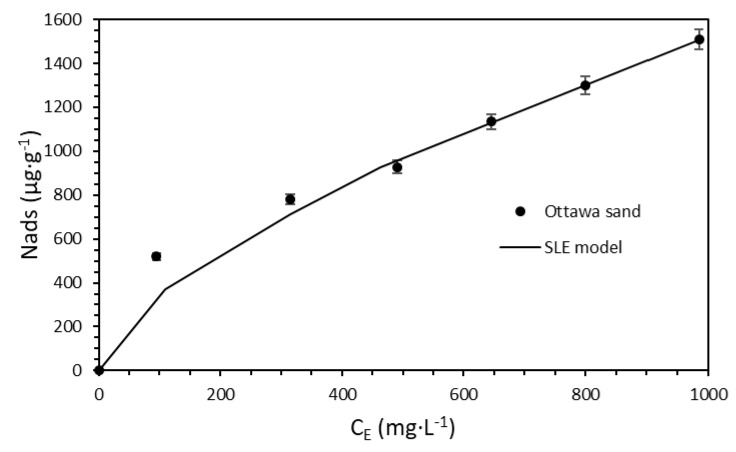
Adsorption isotherms constructed at a constant brine TDS of 0.75% with solutions prepared at 50 °C. The tests were performed using the thermogravimetric method with the SA system in the absence of nanoparticles and varying the surfactant concentration (100, 500, and 1000 mg·L^−1^). The symbols represent the experimental data, while error bars represent standard deviation.

The adsorption of the surfactant onto porous media is critical during EOR operations involving surfactant flooding, as this phenomenon reduces the availability of the tensoactive agent, thereby reducing its interaction with the aqueous phase/crude oil interphase and further hindering the formation of the microemulsion phase [32,34,89]. Therefore, the surfactant adsorption on sandstone was studied using a thermogravimetric method with Ottawa sand grains used as adsorbent and varying the surfactant concentration (100, 500, and 1000 mg·L^−1^). A Type I(a) adsorption isotherm was observed (Figure 10), according to the IUPAC classification [90]. The adsorption isotherm shape may be related to the formation of surfactant micelles due to the presence of dissolved ions in the brine, which limits the adsorption to the sandstone grain surface to just mere molecules in low amounts instead of surfactant aggregates [36]. Nonetheless, the high sweep extension in the porous media positions the surfactant near a large rock surface area, which would increase its loss due to adsorption. In this regard, nanoparticle behavior in surfactant adsorption is of great importance as it reduces the tensoactive agent interaction with the rock and maintains its interaction with the brine/oil interphase.

Based on these results, we decided to evaluate the surfactant adsorption onto the nanoparticle surfaces. In this way, the adsorptive capacity of the CNA nanoparticles was determined by varying the nanoparticle concentrations (50, 100, and 300 mg·L^−1^) in dispersions with a fixed concentration of 1000 mg·L^−1^ of the SA surfactant.

**Figure 11:**
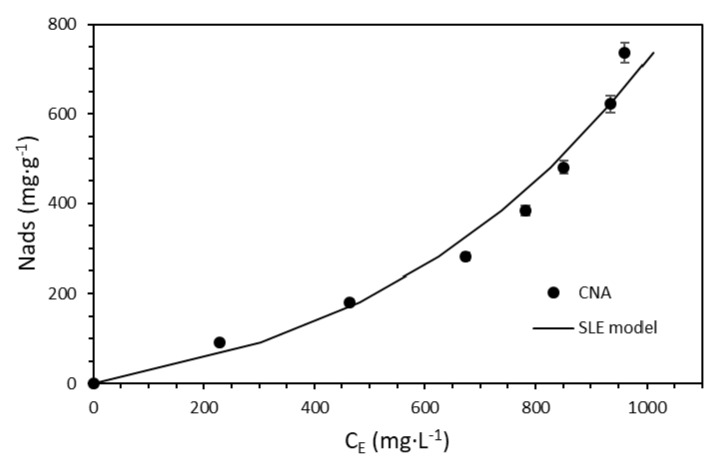
Adsorption isotherms constructed for a constant brine TDS of 0.75% with solutions prepared at 50 °C. The tests were performed using the thermogravimetric method with the SA/CNA system at a fixed concentration of the surfactant of 1000 mg·L^−1^ and varying the nanoparticle concentrations to 50, 100, and 300 mg·L^−1^. The symbols represent the experimental data, while error bars represent standard deviation.

The measured isotherm (Figure 11) is related to a typical Type III shape, which describes a low affinity between the adsorbate and adsorbent [90]. As mentioned before, the isoelectric point of the CNA nanoparticles was at a pH of 2, which means that these would be stable at the brine pH (7.1) due to repulsive forces between nanoparticles caused by the negative charges on a pH above their isoelectric point [76]. However, this behavior could also decrease the nanomaterial affinity for the surfactant, as the negative charges of the CNA produce repulsive forces with the anions of sulfonate groups in the surfactants such as the -SO_2_-OH, which generates a low amount of adsorbed surfactant at low nanoparticles concentrations [47]. Combining this with the dissolved ion availability in the brine due to the dispersion preparation order would enhance the surfactant–surfactant interactions and the formation of micelles, which suggests that the surfactant is adsorbed as aggregates or micelles onto the nanomaterial surfaces rather than as single molecules [84].

Nonetheless, the surfactant adsorption was much higher for the evaluated nanoparticle concentrations, which would partially avoid its retention on the porous media. Therefore, the tentative reduction in the surfactant adsorption onto the rock surface was calculated by subtracting the surfactant adsorbed onto nanoparticles from the adsorbed base amount of the surfactant represented in Figure 10. A total reduction yielding 40% was observed for a fixed surfactant concentration of 1000 mg·L^−1^ and a CNA material concentration of 100 mg·L^−1^. These results indicate that the nanofluid formulation would maintain the near ultra-low IFT of the aqueous phase/crude oil system under reservoir conditions by means of the reduction in the surfactant due to its interaction with the rock.

The solid–liquid equilibrium (SLE) model can describe the adsorptive behavior of the Ottawa sand and the CNA nanoparticles. The SLE model has been widely used in previous studies in which it has been demonstrated as suitable for describing the adsorption behavior of compounds with self-assembly characteristics [60,76,91]. The SLE model has, therefore, been useful in describing the adsorptive behavior of surfactants onto nanoparticles’ surface [45,47,84].

The SLE model includes three distinctive parameters. The first is Henry’s constant (*H*), which accounts for the adsorption affinity of the adsorbate onto the adsorbent solid surface. The lower the *H* parameter value, the higher the adsorbate affinity for adsorption onto a specific surface. The *K* constant indicates self-assembly of the adsorbate molecules once the primary active sites of the adsorbent surface have been saturated. The final parameter is *N_m_*, which represents the highest adsorptive capacity of the adsorbent, i.e., when the active sites of the adsorbent are completely saturated, and no additional adsorbate layers are placed to surround the adsorbent [60]. In this regard, the SLE model was satisfactory to account for the surfactant–adsorbent interactions.

Table 6 shows the estimated SLE parameters. As expected, the *H* parameter showed a higher affinity for surfactant adsorption onto the Ottawa sand than CNA nanoparticles. This result was expected from the isotherm shapes, as Type III isotherms (CNA nanoparticles) describe a low affinity between the adsorbate and the adsorbent [47]. Moreover, the parameter *K* values followed the order of CNA nanoparticles > Ottawa sand, which was consistent with the isotherm results, as higher *K* values indicate the adsorption of adsorbate aggregates instead of individual molecules, as stated for the CNA nanoparticles [47]. Finally, the *N_m_* followed the order of Ottawa sand > CNA nanoparticles, which was also consistent with the experimental results.

### 3.4. Capillary Number Estimation

**Figure 12:**
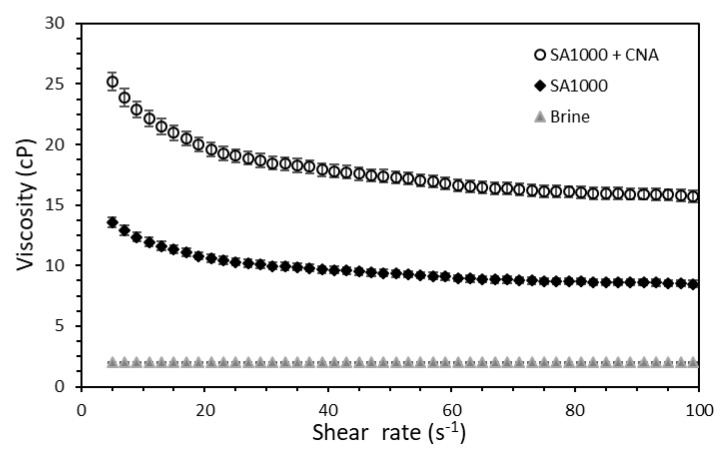
Rheological behavior (viscosity vs. shear rate) for brine and the dispersions in the absence and presence of the evaluated nanoparticles at a fixed concentration of 100 mg∙L^−1^ measured at 50 °C.

The rheological behavior and contact angle of the brine, SA1000, and SA1000 with the fixed concentration of 100 mg·L^−1^ of the CNA nanoparticles, were estimated to obtain their capillary number values. The rheological behavior (viscosity vs. shear rate) of the mentioned samples is presented in Figure 12. Typically, surfactant inclusion in aqueous solutions below the CMC values does not noticeably affect its viscosity due to the formation of single-molecule dispersions [92]. However, the formation of the micelles when surfactant concentrations are higher than the CMC changes the brine microstructure and tends to increase its viscosity as surfactant aggregation increases [93]. Moreover, it is well known that nanofluids formed as suspensions of nanoparticles in a carrier fluid increase whole-system viscosity due to the addition of solid particles [12]. In this way, the nanoparticle–surfactant assembly was expected to increase the viscosity of the aqueous solution.

The sample viscosity followed the order of SA1000 + CNA > SA1000 > brine for all of the shear rate range, which was mainly due to the presence of surfactants and nanoparticles in the first two samples. The brine viscosity was observed not to be significantly dependent on the applied shear rate, which means that it exhibited approximate Newtonian behavior regardless of the presence of the dissolved solids. Conversely, the dispersions in the absence and presence of the nanoparticulated systems had a typical shear-thinning behavior, which was more pronounced at low shear rates in which the fluid deformation was also lower.

**Figure 13:**
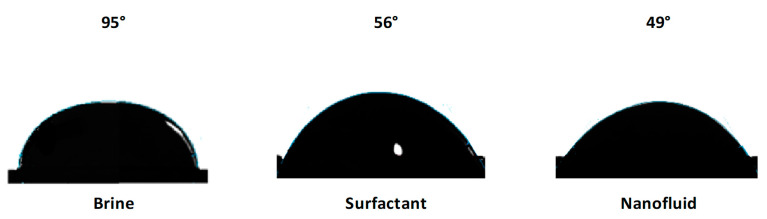
Contact angle measurements of the water phase using the brine and the dispersions in the absence and presence of the evaluated nanoparticles at a fixed concentration of 100 mg∙L^−1^ as treatments. The experiments were performed at 50 °C.

The contact angle results for the water phase are shown in Figure 13. From previous research, it is well known that a contact that tends to 0° denotes a surface preference by the evaluated phase, while above 90° indicates non-wettable behavior or preference [9]. Using the sole brine as treatment had no significant effects on the surface preference by water, as the measured contact angle was approximately 95°. However, both the surfactant and the nanofluid significantly affected the wettability alteration of the used cores, obtaining contact angles of 56 and 49°, respectively. This result was mainly due to the surfactant interaction with the rock surface, which was surrounded by the deposited asphaltenes, and this asphaltene–surfactant interaction exposed the hydrophilic tail of the surfactant, making feasible its interaction with water [94,95]. On the other hand, the inclusion of nanoparticles in the medium had a positive effect in reverting the rock preference from an oil-wet to a water-wet state as reported in other studies [9]. The main mechanisms governing this behavior is linked with a sort of decoration of the nanoparticles in the porous medium, and as the nanoparticles have high polarity due to the hydroxyl groups (O-H) on their surface [61], their interaction with water is also enhanced.

With the contact angle and viscosity results, it was expected that the capillary number value would follow the order of brine < surfactant < nanofluid.

**Figure 14:**
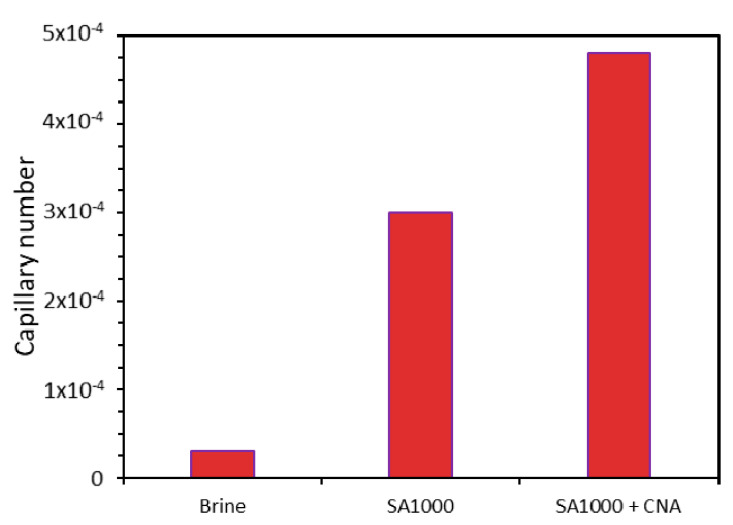
Capillary number (CN) estimated at a shear rate of 7 s^−1^ and the respective viscosity of the brine and dispersions in the absence and presence of the evaluated nanoparticles at a fixed concentration of 100 mg∙L^−1^.

The capillary number was estimated according to equation 2 at a shear rate of 7.0 s^−1^ and the respective viscosity of the brine and dispersions in the absence and presence of the evaluated nanoparticles at a fixed concentration of 100 mg∙L^−1^, as it was a reference value of the flow velocity of the fluids under reservoir (in-depth) conditions [12]. The obtained results (Figure 14) were as expected: the capillary number followed the order brine < surfactant < nanofluid with values of 3 × 10^−5^, 3 × 10^−4^, and 5 × 10^−4^, respectively. The performance was improved when the nanofluid, instead of just surfactant, was included, regardless of the IFT similarity. This result was due to its higher viscosity values and, to a lesser degree, the slight difference obtained in the contact angle experiments.

The capillary number increased by 900% and 1500% when using the dispersion and the nanofluid, respectively. The nanoparticle–surfactant assembly exhibited a synergistic phenomenon, as the capillary number of the joint system experienced a higher increase than the individual dispersion (surfactant–brine) and also to the single nanoparticle usage, which in previous studies has been observed to be approximately 600% [12].

### 3.5. Coreflooding Tests

The dynamic experiments carried out under reservoir conditions through coreflooding are of particular interest as these provide representative data for determining the feasibility of the technology upscaling to the field trial. In this sense, core displacement tests (1) determined possible injectivity changes towards nanofluid usage and (2) evaluated the increase in the tertiary recovery crude oil due to the surfactant flooding enhancement in the presence of nanoparticles.

**Figure 15:**
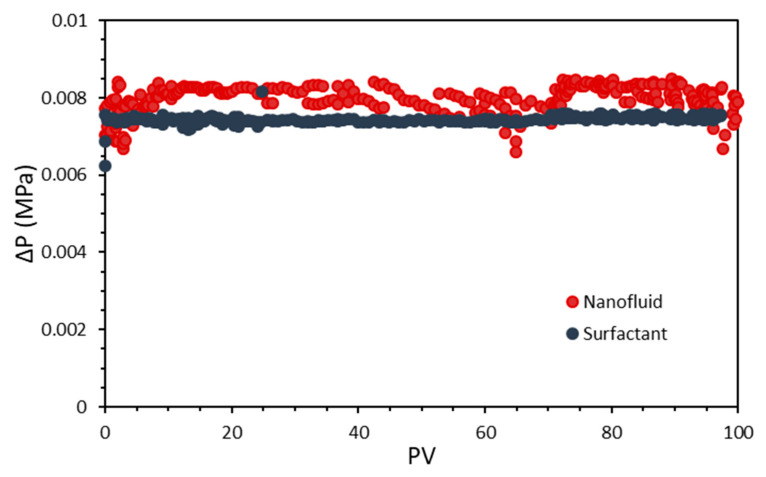
Injectivity tests carried out for the surfactant and nanofluid formulations under reservoir conditions (50 °C, and 9.31 and 3.45 MPa of overburden and back pressure, respectively). For the surfactant injection, the Core 1 reported in Table 2 was used, while the Core 2 was implemented for the nanofluid injection.

The injectivity test results are shown in Figure 15. The experiments were conducted for the surfactant and nanofluid formulations under reservoir conditions (50 °C, and 9.31 and 3.45 MPa of overburden and back pressure, respectively). For the surfactant injection, the Core 1 reported in Table 2 was used, while the Core 2 was implemented for the nanofluid injection. A constant pressure drop (ΔP) was observed for the surfactant injection with a value yielding 0.0076 MPa. The nanofluid injection followed a similar trend with a ΔP below 0.0083 MPa, and slightly more fluctuation, which was more pronounced below the first 10 PVs injected. This tendency could be derived from the surfactant adsorption onto the nanoparticle surfaces, which causes the entire nanoparticle–surfactant assembly to increase in size slightly [95]. Nonetheless, in general terms, no adverse effects on the fluid injectivity were observed.

The presence of nanoparticles in surfactant dispersions positively affects the injected fluid viscosity, wettability alteration, and IFT maintenance and/or reduction [46]. Conventionally, during improved water injection with surfactants present, crude oil recovery is enhanced. This enhancement is accomplished by avoiding the displacement front fingering and displacing the trapped crude oil by decreasing the capillary forces that hinder crude oil mobilization and/or increasing the displacement fluid properties, which are represented in Nc [8,18,20]. Furthermore, nanoparticle inclusion plays an essential role in the improvement of displacement fluids such as by the viscosity, wettability alteration, and the IFT.

By avoiding the surfactant loss due to its adsorption phenomena, the nanoparticles at least maintain the IFT values. However, the nanoparticle–surfactant assembly also can interact with the brine/oil interphase, and in this way, the adsorbed surfactant on the nanoparticle surfaces would still have a major role in the displacement performance [45,47]. The main mechanism consists of the targeted deliverability of the surfactant due to the ability of the nanoparticles to transport the surfactant to the oil–water interphase [12,44]. In this way, the fluid injection, including nanoparticles and surfactants, would increase the sweep efficiency by decreasing the fingering of the displacement front, as demonstrated in other studies [96].

**Figure 16:**
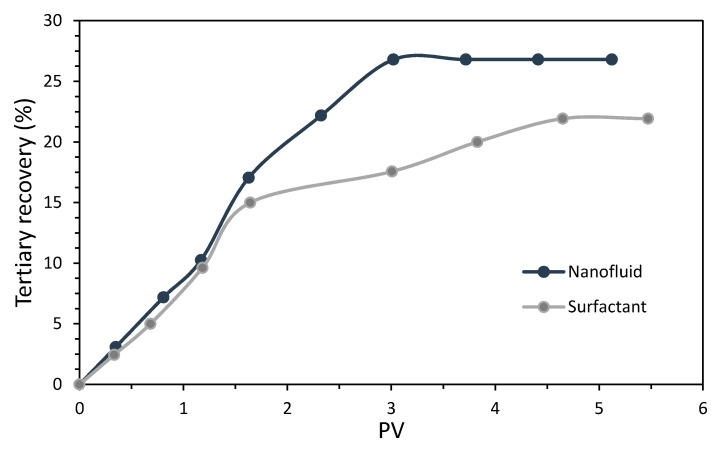
Tertiary recovery estimated for separate injections of the surfactant and nanofluid under reservoir conditions (50 °C, and 9.31 and 3.45 MPa of overburden and back pressure, respectively).

The recovery factors estimated for the surfactant and nanofluid injection are presented in Figure 16. There was a similar initial slope for both curves. However, after the injection of 1.5 PVs, a differentiated path for the surfactant and nanofluid recovery curves was observed with a higher slope for the latter. This difference in curve shapes was related to the surfactant adsorption on the porous media in the absence of nanoparticles, which decreased the IFT. The use of nanoparticles partially avoided this surfactant loss, which resulted in a 5% difference of tertiary recovery (3% of the OOIP) upon the injection of 3 PVs of the displacement fluid. This higher difference was gradually reduced until 4.5 PVs, presumably due to saturation of the rock active sites with the surfactant. Although, at maximum recovery values, we observed a tertiary crude oil recovery increase for the 18% nanofluid compared to the sole surfactant injection, which represented 5% of the OOIP. This enhanced performance for the displacement fluid in the presence of nanoparticles was also narrowly related to the superior performance obtained by the nanofluid regarding the capillary number, which involved a greater wettability alteration of the porous media, and a higher displacement fluid viscosity.

As for the Nc, a synergistic process occurred when using the nanoparticles–surfactants assembly, as a higher efficiency in the oil recovery was obtained compared to the separated elements that composed the nanofluid, including the individual nanoparticles which in a previous study were shown to provide no additional recovery at 100 mg·L^−1^.

The field trial application was conducted, commencing with the nanofluid formulation, including 1000 mg·L^−1^ of the SA surfactant.

### 3.6. Field Application

Field application is straightforward, performed only with a simple injection pump, instead of more complicated surfactant injection facilities [97]. For both patterns, the initial nanofluid formulation included 1000 and 100 mg·L^−1^ of the SA surfactant and CNA nanoparticles, respectively. The surfactant concentration was gradually decreased to 350–400 mg·L^−1^ for both patterns to maintain the IFT of the aqueous phase/crude oil interphase in the LIFT region with the assistance of nanoparticles, and thereby, developing a cost-effective process.

The decline rate value and the sum of production of both patterns were estimated obeying an exponential model represented by the following equations:

(7) $$D=\frac{\Delta q/\bar{q}}{\Delta t}$$

(8) $$q=q_{o}e^{\left(-D\cdot \Delta t\right)}$$

(9) $$Np=\frac{qo-q}{D}$$

in which D is the decline rate, Δt and Δq are the time and the oil flow rate difference, $\bar{q}$ is the average flow rate during the evaluated period, and Np is the cumulative oil production for a defined time interval. In this way, the cumulative oil production increment was estimated by subtracting the estimated contribution of each pattern from the total real oil production.

**Figure 17:**
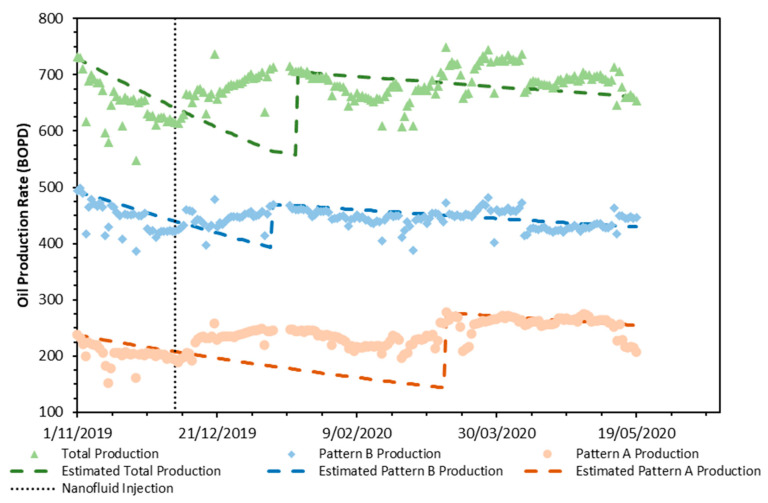
Real oil production rate registered for the patterns A and B and the total pattern of daily production. The symbols denote the respective production rates while the dashed lines represent production decline.

The oil production rate was monitored during the entire field test period (Figure 17), and nearly seven days after the nanofluid injection started, an increment of oil production was obtained for Pattern A, which was reflected in the total pattern production. On the other hand, for pattern B, a substantial incremental production was not observed until 17 days after the field test started. This behavior was attributable to all the wells connected in pattern A with a positive response upon the nanofluid injection, while for pattern B, approximately six wells did not have an appreciable response to the enhanced displacement fluid. This last result was presumably related to an inherent damage formation process not associated with the nanofluid injection.

Moreover, for the first month after commencing the field test, a total mean production rate increase of 78 BOPD was estimated, which slightly decreased due to the surfactant concentration reduction in the injected fluid. The surfactant dosage decreases in the injected nanofluid did not produce substantial changes in the production rate. Thus, it is concluded that it is feasible to perform the EOR operation with an acceptable cost-effective margin. A visual change in the oil production decline rate was observed for both patterns A and B, which was reflected in the total oil production, as this value changed from −0.104 to −0.016 month^−1^ after the intervention. This last result was associated with a displacement sweep efficiency increase which presumably, would positively affect the incorporation of long-term oil reserves.

Finally, at the end of the pattern production tracing (May 19), the production rate was 660 BOPD, which represented an increase of 45 BOPD compared to the production rate before conducting the nanofluid injection.

**Figure 18:**
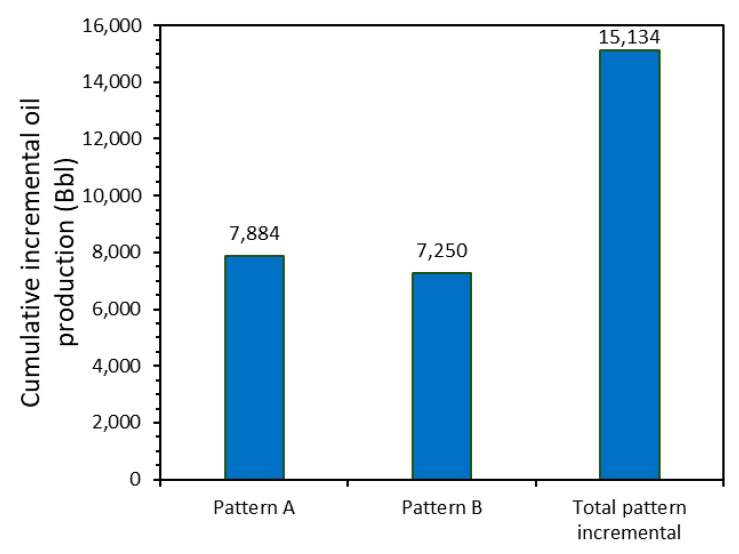
Cumulative incremental oil production obtained during the field tests for the patterns A and B and the total pattern.

A summary of the cumulative incremental oil production for patterns A and B is seen in Figure 18. During the different field test stages, there was an incremental cumulative production of 13,382 and 16,654 Bbls for patterns A and B, respectively, which represented a total cumulative incremental production of 30,035 Bbls. Although pattern B had a higher oil production rate at the beginning of the operation, and thus a much higher production response was expected for this pattern than A, the overall performances were similar. This observation could be related to the lack of connectivity between some wells with the injector of pattern B, as discussed previously. Thus, it is expected that intervention for the damage formation remediation in these specific wells would lead to much more promising results in production.

## 4. Conclusions

We present here the first application worldwide of nanotechnology in an enhanced oil recovery process under real field conditions. The upscaling for the application of an enhanced surfactant injection with nanotechnology in a field trial was appropriately evaluated and described through the surfactant–nanoparticle–brine tuning process, which included an assessment of the surfactant and nanoparticle type and concentration, as well as the amount of total dissolved solids (TDS) in the brine.

The first steps of the tuning process showed that the IFT is narrowly related to the presence of dissolved ions in the brine as these tend to decrease the repulsive forces between surfactants facilitating the brine/oil interphase saturation with the surfactant molecules. In this regard, the best results were obtained with 0.75% TDS in the brine formulation, while the SA surfactant had the best response both in the absence and presence of nanoparticles. Thus, the best nanofluid formulation was that obtained with the SA/CNA combination as it exhibited a synergistic effect in which the IFT values were maintained and/or reduced. Moreover, the surfactant and nanoparticle concentration evaluation showed that for concentrations near the surfactant CMC, the nanoparticle addition increased the IFT rather than decreasing or maintaining it due to the surfactant adsorption on the nanoparticle surface. In this way, we observed a synergistic process between the surfactant and nanoparticles for a SA concentration of 750 mg∙L^−1^ and above, while the nanomaterial dosage was established at a fixed 100 mg∙L^−1^. Furthermore, the adsorption tests revealed that the surfactant adsorption on the rock was reduced by at least 40% due to the presence of nanoparticles. This phenomenon may help maintain the IFT as the surfactant–nanoparticles assembly can move and interact with the aqueous phase/crude oil interphase as well as the free surfactant in the porous media.

Furthermore, the capillary number was estimated for the brine, the sole surfactant, and the nanofluid, obtaining values of 3×10^−5^, 3×10^−4^, and 5×10^−4^, respectively. This behavior was attributed to the viscosity increase in the dispersion in the presence of nanoparticles and, to a minor degree, the wettability alteration of the porous media. Thereby, a better performance in core flooding tests was expected for the nanofluid. Indeed, the nanofluid performed better in the displacement tests compared to the sole surfactant usage with a tertiary recovery increase of almost 18%. From the shape of the curve, it was also concluded that under dynamic conditions, the nanoparticles maintained the IFT value by avoiding the surfactant adsorption on the rock, and therefore, increased the recovery performance.

The field test was conducted in two previously characterized displacement patterns (patterns A and B) of a Colombian field. First, a nanofluid formulation of 1000 and 100 mg∙L^−1^ of the SA surfactant and CNA nanoparticles were used, respectively, with a gradual surfactant dosage reduction throughout the field test application. For the first month, there was a total estimated incremental oil rate production of 78 BOPD, with a minor decrease towards the final stage of the field test attributed to the surfactant dosage decrease to 400–350 mg∙L^−1^. By May 19, a total cumulative production incremental of 30035 Bbls from both patterns was estimated with several positive changes in the production decline rate, which changed from −0.104 to −0.016 month^−1^ after the intervention.

## Figures and Tables

**Table 1:** Synthetic brine formulation resembling the salt composition of the on-site injected brine.

Salts	Concentration (mg·L^−1^)
NaCl	5.717
CaCl_2_.2H_2_O	13.525
MgCl_2_.6H_2_O	0.3833
BaCl_2_.2H_2_O	0.0996
KCl	7.4121

**Table 2:** Properties of the porous media used for the surfactant and nanofluid injection.

Porous Media Properties		
Property	Core 1	Core 2
Length (cm)	7.1	17.5
Diameter (cm)	3.8	4.4
Porosity (%)	16	17
Porous volume (cm^3^)	13	43.5
Mineralogy	50% Ottawa sand−50% reservoir core cuts	

**Table 3:** Nanofluid flooding stage injection as a function of the surfactant concentration in the displacement fluid.

Surfactant Concentration (mg·L^−1^)	Total Treatment Injected (gal/d)	Date
**Pattern A**		
1000	54.6	6-Dec-19
500	48	23-Dec-19
300	29	8-Jan-20
350	33	28-Jan-20
350	31	3-Mar-20
400	37	20-Mar-20
**Pattern B**		
1000	126	6-Dec-19
500	76	23-Dec-19
300	46	8-Jan-20
350	52	28-Jan-20
350	50	6-Apr-20

**Table 4:** Density, critical micelle concentration (CMC), and hydrophilic–lipophilic balance (HLB) for the two different mixtures of commercial anionic surfactants (SA and SB).

Surfactant	Density (g·L^−1^)	CMC (mg·L^−1^)	HLB
SA	0.94	250	11
SB	0.98	300	11

**Table 5:** Nanoparticle mean particle size (dp_50_) measured by the dynamic light scattering (DLS) technique, surface area (S_BET_) obtained by N_2_ physisorption, and isoelectric point (pH_IEP_).

Particle	dp50 (± 1 nm)	S_BET_ (± 1 m^2^∙g^−1^)	pH_IEP_ ± 0.1
CNA	71	192	2.0
CNB	70	221	2.2

**Table 6:** Parameters of the solid–liquid equilibrium (SLE) model calculated for Ottawa sand and CNA adsorption isotherms at a temperature of 50 °C.

Adsorbent	H (mg·g^−1^)	K (g·g^−1^)	N_m_ (g·g^−1^)	RSME (%)
Ottawa sand	0.00039	1.75 × 10^−6^	6042.51	4.58
CNA	5.16	2.64	4.22	4.21
